# Absence of neurological abnormalities in mice homozygous for the *Polr3a* G672E hypomyelinating leukodystrophy mutation

**DOI:** 10.1186/s13041-017-0294-y

**Published:** 2017-04-13

**Authors:** Karine Choquet, Sharon Yang, Robyn D. Moir, Diane Forget, Roxanne Larivière, Annie Bouchard, Christian Poitras, Nicolas Sgarioto, Marie-Josée Dicaire, Forough Noohi, Timothy E. Kennedy, Joseph Rochford, Geneviève Bernard, Martin Teichmann, Benoit Coulombe, Ian M. Willis, Claudia L. Kleinman, Bernard Brais

**Affiliations:** 1 Montreal Neurological Institute, McGill University, 3801 University Street, room 622, Montréal, Québec H3A 2B4 Canada; 2 Department of Human Genetics, McGill University, Montréal, Québec Canada; 3 Lady Davis Institute for Medical Research, Jewish General Hospital, Montréal, Québec Canada; 4 Department of Biochemistry, Albert Einstein College of Medicine, Bronx, New York USA; 5 Translational Proteomics Laboratory, Institut de recherches cliniques de Montréal (IRCM), Montréal, Québec Canada; 6 Douglas Institute Research Center, Montréal, Québec Canada; 7 Departments of Neurology and Neurosurgery, and Pediatrics, McGill University, Montreal, Canada; 8 Department of Medical Genetics, Montreal Children’s Hospital, McGill University Health Center, Montreal, Canada; 9 Child Health and Human Development Program, Research Institute of the McGill University Health Center, Montreal, Canada; 10 INSERM U1212 – CNRS UMR5320, Université de Bordeaux, Bordeaux, France; 11 Département de biochimie et médecine moléculaire, Université de Montréal, Montréal, Québec Canada

**Keywords:** Leukodystrophy, *POLR3A*, Mouse model, Hypomyelination, RNA Polymerase III, Transfer RNAs

## Abstract

Recessive mutations in the ubiquitously expressed *POLR3A* gene cause one of the most frequent forms of childhood-onset hypomyelinating leukodystrophy (HLD): POLR3-HLD. *POLR3A* encodes the largest subunit of RNA Polymerase III (Pol III), which is responsible for the transcription of transfer RNAs (tRNAs) and a large array of other small non-coding RNAs. In order to study the central nervous system pathophysiology of the disease, we introduced the French Canadian founder *Polr3a* mutation c.2015G > A (p.G672E) in mice, generating homozygous knock-in (KI/KI) as well as compound heterozygous mice for one *Polr3a* KI and one null allele (KI/KO). Both KI/KI and KI/KO mice are viable and are able to reproduce. To establish if they manifest a motor phenotype, WT, KI/KI and KI/KO mice were submitted to a battery of behavioral tests over one year. The KI/KI and KI/KO mice have overall normal balance, muscle strength and general locomotion. Cerebral and cerebellar Luxol Fast Blue staining and measurement of levels of myelin proteins showed no significant differences between the three groups, suggesting that myelination is not overtly impaired in *Polr3a* KI/KI and KI/KO mice. Finally, expression levels of several Pol III transcripts in the brain showed no statistically significant differences. We conclude that the first transgenic mice with a leukodystrophy-causing *Polr3a* mutation do not recapitulate the childhood-onset HLD observed in the majority of human patients with *POLR3A* mutations, and provide essential information to guide selection of *Polr3a* mutations for developing future mouse models of the disease.

**Electronic supplementary material:**

The online version of this article (doi:10.1186/s13041-017-0294-y) contains supplementary material, which is available to authorized users.

## Background

Hypomyelinating leukodystrophies are a heterogeneous group of neurodegenerative diseases characterized by impaired cerebral myelin formation. POLR3-related hypomyelinating leukodystrophy (POLR3-HLD), also called 4H leukodystrophy, is caused by recessive mutations in *POLR3A*, *POLR3B* or *POLR1C* [1–4]. Patients usually present in early childhood or adolescence with motor regression, cerebellar features and/or cognitive dysfunction [1, 5]. In many cases, they also display hypogonadotropic hypogonadism and/or hypodontia [1, 5]. Diffuse hypomyelination with relative preservation (T2 hypointensity) of myelination of the dentate nuclei, anterolateral nuclei of the thalami, globi pallidi and optic radiations, as well as a thin corpus callosum and cerebellar atrophy are observed on magnetic resonance imaging (MRI) in the majority of POLR3-mutated patients [5–8].

*POLR3A*, *POLR3B* and *POLR1C* encode subunits of RNA Polymerase III (Pol III), one of the three essential eukaryotic RNA polymerases. Specifically, Pol III is responsible for the synthesis of several types of non-coding RNAs (ncRNAs), including transfer RNAs (tRNAs), 5S ribosomal RNA (rRNA), U6 small nuclear RNA and BC200 RNA [9]. Pol III is a large enzymatic complex composed of 17 subunits. POLR3A and POLR3B, the two largest subunits, form the catalytic center of the enzyme.

Since the initial identification of mutations in *POLR3A* [1], more than 100 mutations in *POLR3A*, *POLR3B* and *POLR1C* have been identified in over 130 patients with POLR3-HLD [1–5, 8, 10–19]. The majority of mutations are private or present in only a handful of patients [5]. While most international POLR3-HLD patients are compound heterozygotes, the majority of French Canadian cases are homozygous for the c.2015G > A (p.G672E) mutation in *POLR3A*, suggesting a founder effect in this population [1, 5]. In addition to this genetic heterogeneity, POLR3-HLD is characterized by important inter- and rarely intra-familial clinical variability, both in symptoms and severity, and its phenotypic spectrum continues to expand [8, 20, 21]. Notably, two recent studies described patients with cerebellar atrophy only [8] or with involvement of the striatum and red nuclei but normally myelinated white matter [21], suggesting that diffuse hypomyelination is not an obligate feature of the disorder [8].

Despite the major advances in the clinical and genetic characterization of POLR3-HLD, the molecular basis of its pathophysiology remains poorly understood. Mutations are located throughout the three genes and are likely to impact different functional aspects of Pol III, which would in all cases lead to enzyme hypofunction and decreased expression of ncRNAs synthesized by Pol III [1–3, 22]. Indeed, a recent study using FLAG-tagged POLR1C mutants transfected in HeLa cells demonstrated that two *POLR1C* missense mutations cause impaired assembly of the Pol III complex, accumulation of the mutated subunits in the cytoplasm and reduced Pol III occupancy at its target promoters, suggesting decreased transcription of the corresponding genes [3]. In addition, overexpression of missense alleles of *Rpc1*, the yeast ortholog of *POLR3A,* in *S. pombe*, led to reduced precursor tRNA levels, a proxy for transcription, and changes in tRNA modification and translation fidelity [23]. A key question is how mutations in such an essential and ubiquitously expressed enzymatic complex lead to a central nervous system (CNS)-specific disease. Most Pol III transcripts are ubiquitously expressed and several of them are at their highest expression level in the CNS [24, 25]. Moreover, POLR3-HLD belongs to a growing number of neurological diseases, including several leukodystrophies, caused by mutations in genes that are also related to tRNA biology [26–32], suggesting that impaired tRNA biogenesis could be particularly detrimental to the CNS.

In this study, we generated and characterized a knock-in (KI) mouse model carrying the common French Canadian *Polr3a* c.2015G > A (p.G672E) mutation in order to determine if it recapitulates POLR3-HLD features. Herein, we describe the results from a yearlong study of motor function in this first transgenic exploratory model of POLR3-HLD, as well as its molecular and histological characterization.

## Results

### Generation of *Polr3a* KI/KI and KI/KO mouse models

To obtain a relevant model of POLR3-HLD, we generated a KI mouse carrying the c.2015G > A (p.G672E) mutation in *Polr3a*, a mutation chosen based on its frequency in French Canadian cases and on the report of several human homozygous cases [1, 5]. Indeed, we obtained viable KI/KI mice and confirmed the expression of the homozygous c.2015G > A (p.G672E) mutation in these animals by Sanger sequencing of tail genomic DNA as well as brain cDNA (Additional file 1: Figure S1A). We also generated a compound heterozygous *Polr3a* mouse line carrying one KI allele and one null allele (KI/KO). Heterozygous *Polr3a* knockout (KO) mice were produced by insertion of a gene trap cassette in intron 21. The portion of intron 21 upstream of the cassette is retained in the mRNA of these mice, leading to a frameshift and premature stop codon (p.E968VfsX12) (Additional file 1: Figure S2). As expected, homozygous *Polr3a* KO mice are embryonically lethal (Additional file 1: Figure S1B). KI/KI mice were bred with heterozygous *Polr3a* KO mice to create the KI/KO mouse line. Both KI/KI and KI/KO mice reproduce normally and do not display a grossly abnormal phenotype at 12 months of age. At the protein level, full-length POLR3A levels were comparable in the cerebrum of one-year-old KI/KI, KI/KO and WT mice (Additional file 1: Figure S1D). The KO allele is predicted to cause a frameshift leading to premature termination at amino acid 980 and resulting in a protein of approximately 100 kDa. We did not observe a band of that size accumulating in KI/KO mice (Additional file 1: Figure S1D), implying that the KO mRNA and/or protein is rapidly degraded. In addition, the normal levels of full-length protein in KI/KI and KI/KO mice indicate that the G672E mutation does not impair stability of the POLR3A protein.

### Characterization of motor function over one year

**Fig. 1:**
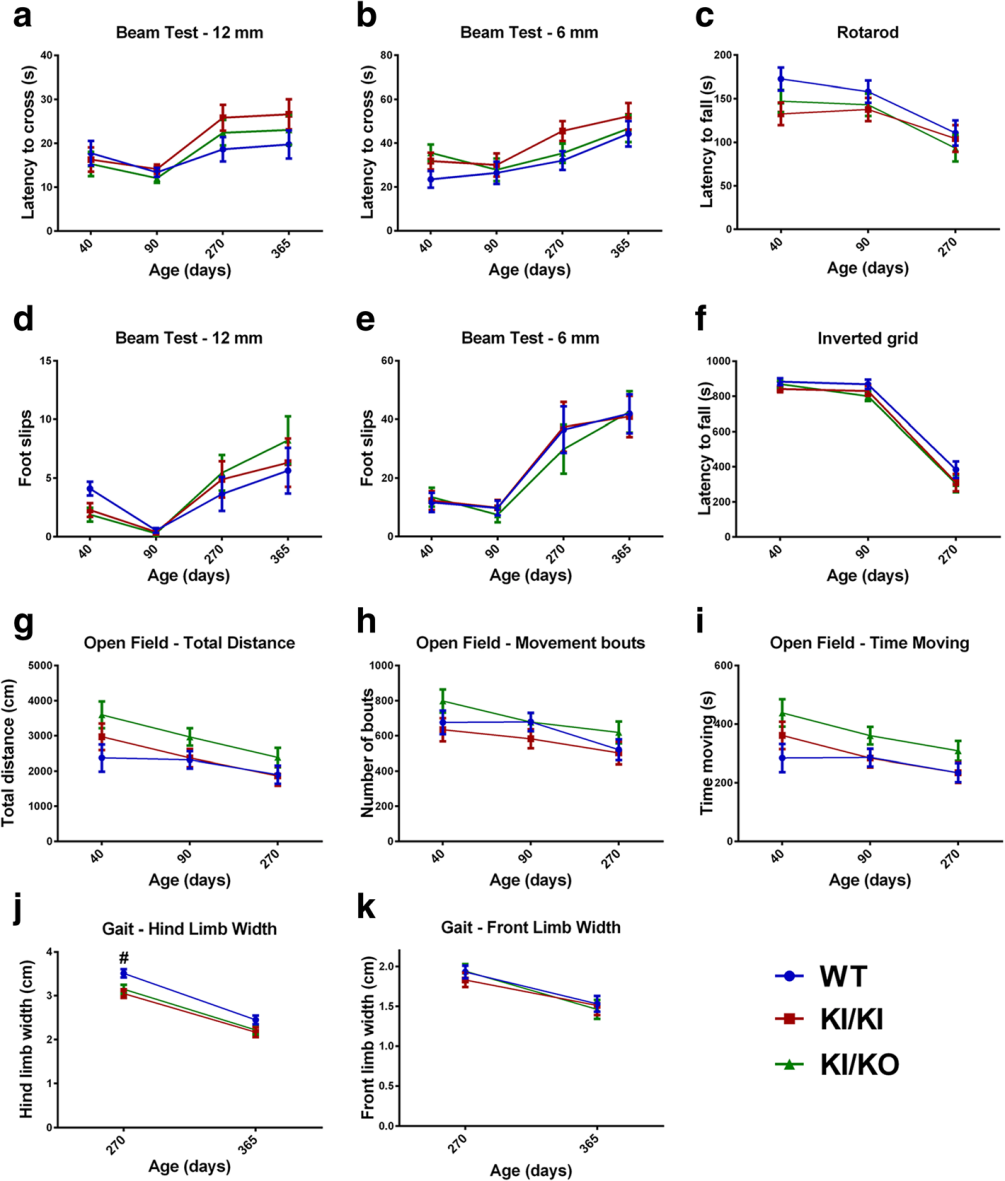
Yearlong study of motor function in *Polr3a* KI/KI and KI/KO mice. Results from the 12 mm (**a**, **d**) and 6 mm (**b**, **e**) beam test at four time points consisting of three trials per mouse. Latencies to cross (**a**, **b**) and number of foot slips (**d**, **e**) were recorded for both beam sizes. **c**, **f**) Results from the rotarod (**c**) and inverted grid (**f**) tests performed at three time points. The rotarod and inverted grid consisted of three trials per mouse. **g**-**i**) Results from the open field test performed at three time points. The open field test was run for 90 min per mouse during which total distance traveled (**g**), number of movements bouts (**h**) and total time spent moving (**i**) were recorded for each 10 min interval. The results represent the sum of all 10 min intervals. **j**-**k**) Results from gait analysis performed at the two latest time points. Paws were covered in color paint and mice were allowed to walk on a white paper-covered narrow runway. Distance between fore limbs and hind limbs was measured. All tests were performed on ≥14 female mice per group. For the beam test, rotarod and inverted grid, data are represented as adjusted least squares means +/− SEM of the sum of the three trials for each group. Groups were compared with one-way ANCOVA for each time point. #: *p <* 0.01

Individuals with *POLR3A* mutations, including those homozygous for the c.2015G > A (p.G672E) mutation, manifest cerebellar and upper motor neuron signs leading to impaired gait, coordination and balance as well as cognitive dysfunction [1]. We thus performed balance beam, rotarod, open field and inverted grid tests to assess balance, coordination, general locomotion, and muscle strength (Fig. 1). Since the body weights of the mice were variable, especially at later time points (Additional file 1: Figure S3), we used one-way analysis of covariance (ANCOVA) with weight as the covariate to compare behavioral measures between genotypes (Fig. 1). At 40 and 90 days old, there were no significant differences between the three groups, implying that *Polr3a* KI/KI and KI/KO mice do not develop an early-onset motor phenotype. While some differences were observed on the beam test at 270 and 365 days old (Additional file 1: Figure S4), those were largely attributable to weight and did not remain after adjustment of the data for this variable (Fig. 1). To complement the beam test, we performed gait analysis (Fig. 1j-ik). Both KI/KI and KI/KO mice displayed a small but statistically significant reduction in their back paws limb width compared to WT mice (*p-*value < 0.01) at 270 days old. The test was repeated at 365 days old and showed the same trend but the difference between groups was not statistically significant (Fig. 1j). This may reflect a very mild phenotype that would require testing of older mice for confirmation. In summary, the extensive panel of tests performed strongly suggests that *Polr3a* KI/KI and KI/KO mice do not display motor dysfunction at one year of age.

### Analysis of myelination and cerebellar integrity

**Fig. 2:**
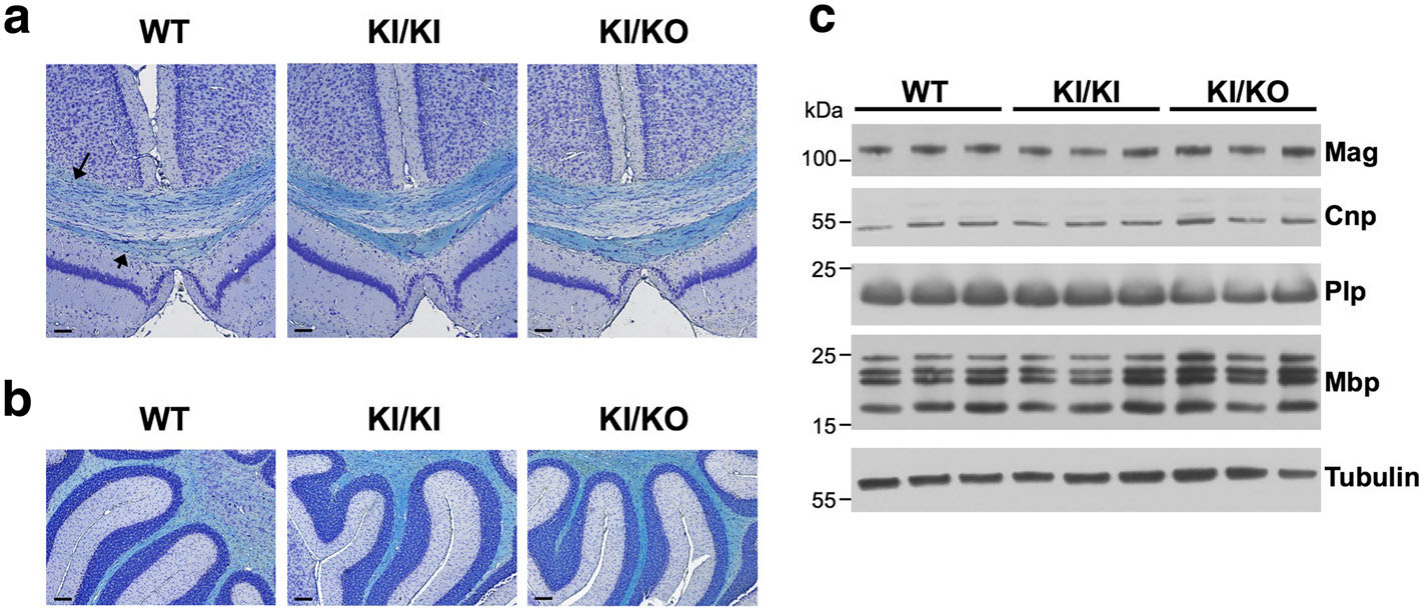
Normal myelination in *Polr3a* KI/KI and KI/KO mice. **a**-**b**) Luxol Fast Blue staining of coronal sections (**a**) showing the corpus callosum (long arrow) and dorsal fornix (short arrow), both myelinated, and of sagittal sections (**b**) of the cerebellum. Staining was performed on three 90 days old mice per group and representative images are shown for each group. Scale bar = 100 μm. **c**) Immunoblots of myelin proteins using total protein extracts from the brain of 90 days old WT, KI/KI and KI/KO mice

**Fig. 3:**
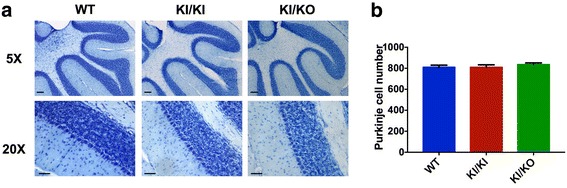
No Purkinje cell loss in *Polr3a* KI/KI and KI/KO mice. **a**) Nissl staining of sagittal cerebellar sections of 365 days old mice. Staining was performed on four mice per group and representative image are shown for each group. Scale bar = 100 μm (top) and 50 μm (bottom). **b**) Purkinje cell counts of mid-sagittal cerebellar sections of 365 days old mice (*n =* 4 per group). Data are represented as mean +/− SEM

Hypomyelination is the main pathological feature of POLR3-HLD [5, 33]. Thus, to assess whether *Polr3a* KI/KI and KI/KO mice display hypomyelination, we stained coronal brain sections from 90 and 365 days old mice with Luxol Fast Blue (LFB), which is commonly used to detect myelin in the CNS. We observed normal and complete myelination in the brain and cerebellum of KI/KI and KI/KO mice, where the staining was indistinguishable from age-matched WT mice (Fig. 2a, b and Additional file 1: Figure S5). In addition, we measured the levels of the major protein components of myelin by western blot in the cerebellum of 90-day-old mice. Protein levels of Myelin Basic Protein (MBP), Proteolipid Protein (PLP), Myelin-associated Glycoprotein (MAG) and 2′,3′-Cyclic Nucleotide 3′ Phosphodiesterase (CNP) were comparable between WT, KI/KI and KI/KO mice (Fig. 2c). These results suggest that *Polr3a* KI/KI and KI/KO mice undergo normal gross myelination and do not experience major demyelination at one year of age. Since cerebellar atrophy and Purkinje cell loss is a major feature in POLR3-HLD [5, 33], we then evaluated cerebellar morphology using Nissl staining followed by Purkinje cell counts in 365-day-old mice. Cerebellar morphology was overall normal (Fig. 3a) as were Purkinje cell numbers (Fig. 3b), implying that KI/KI and KI/KO mice do not present cerebellar atrophy.

### Evaluation of Pol III transcription levels

**Fig. 4:**
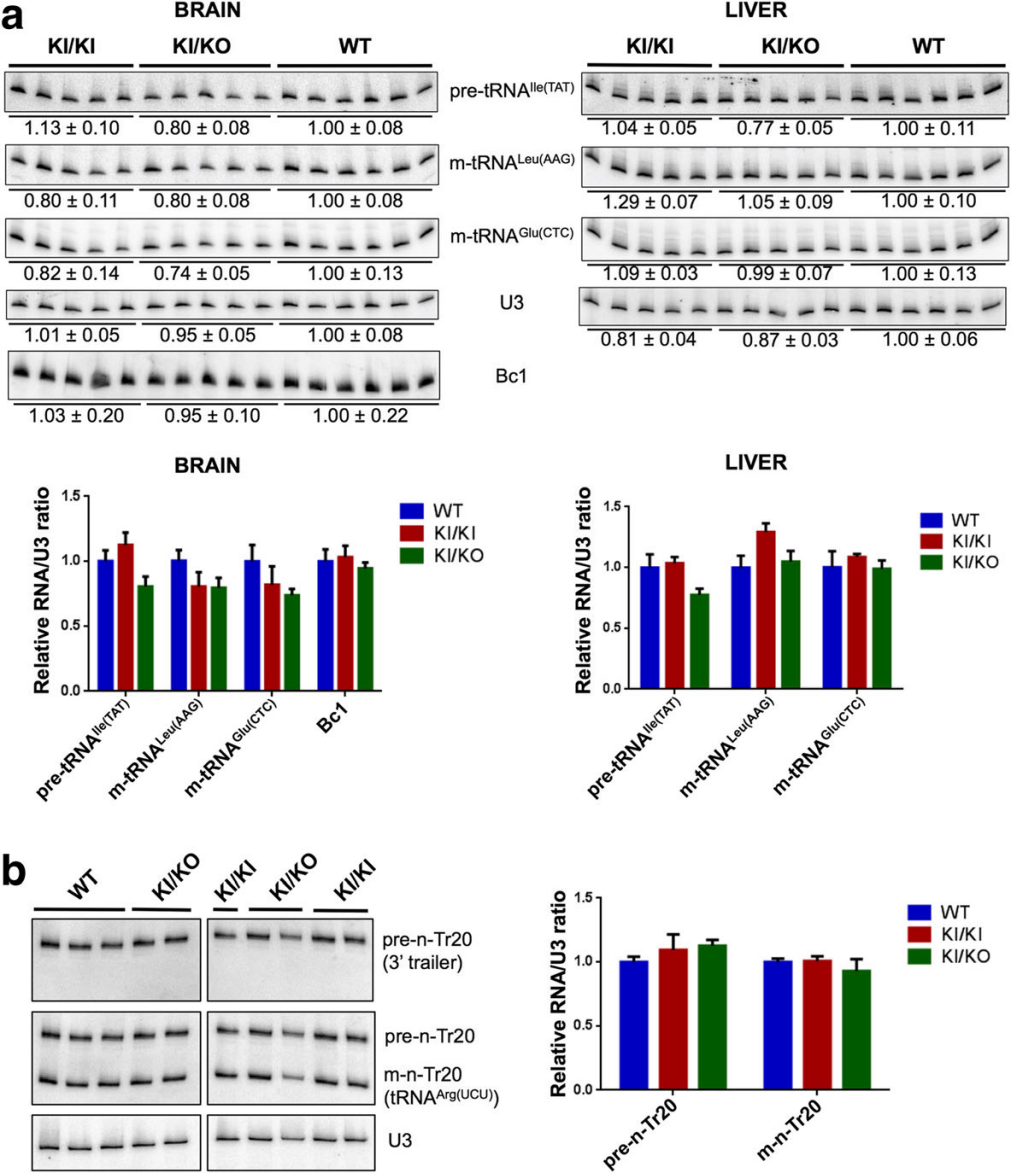
Expression levels of Pol III transcripts in the cerebrum and liver of *Polr3a* KI/KI and KI/KO mice. **a**) Top: Northern blots of precursor (pre) and mature (m) tRNA species from the cerebrum (*left*) and liver (*right*) of 365 days old mice. U3 snRNA was used as a loading control. Bc1 RNA was probed in the cerebrum only. Mean +/− SEM of tRNA or Bc1 levels normalized to U3 snRNA levels are indicated below the blot for each transcript. Bottom: Quantification of Pol III transcripts surveyed by Northern Blot. tRNA levels were normalized to U3 snRNA levels. Data are represented as mean +/− SEM. **b**) Left: Northern blot of precursor (pre) and mature (m) n-Tr20 tRNA^Arg(UCU)^ in the cerebrum of 3-months-old mice, demonstrating low levels of n-Tr20, consistent with these mice having the C57BL/6 J *n-Tr20* genotype (see also Additional file 1: Figure S6B). Right: Quantification of precursor and mature n-Tr20 levels, normalized to U3 snRNA levels

Despite the lack of severe abnormalities at the phenotypic and histological levels, the homozygous c.2015G > A (p.G672E) substitution in *Polr3a* may alter Pol III function. Because of their short half-lives, precursor tRNA levels provide a reliable estimate of Pol III transcription [34–36]. To evaluate the impact of the *Polr3a* G672E mutation on Pol III transcription, we measured the levels of one precursor tRNA and two mature tRNAs in the cerebrum and liver of 90-days-old and one-year-old WT, KI/KI and KI/KO mice (Fig. 4a and Additional file 1: Figure S6). While there were no statistically significant differences in tRNA levels among the three groups, there was a trend towards a small decrease of pre-tRNA^Ile(TAT)^ in one-year-old KI/KO mice (Fig. 4a). We then reasoned that brain-specific transcripts, such as Bc1 RNA and n-Tr20 tRNA [37], might be more sensitive to Pol III mutations. We first confirmed the brain-specific expression of both transcripts (Additional file 1: Figure S6). We then measured the levels of Bc1 RNA, precursor n-Tr20 as well as mature n-Tr20 in the cerebrum of WT, KI/KI and KI/KO mice, but we did not detect differences between groups (Fig. 4 and Additional file 1: Figure S6). Therefore, our results suggest that the *Polr3a* G672E mutation does not significantly impair Pol III transcript levels, although it may result in a minor effect on the transcription of tRNA genes in whole cerebrum of one-year-old *Polr3a* KI/KO hypomorphic mice.

### Impact of the *POLR3A* G672E mutation in human cells

**Fig. 5:**
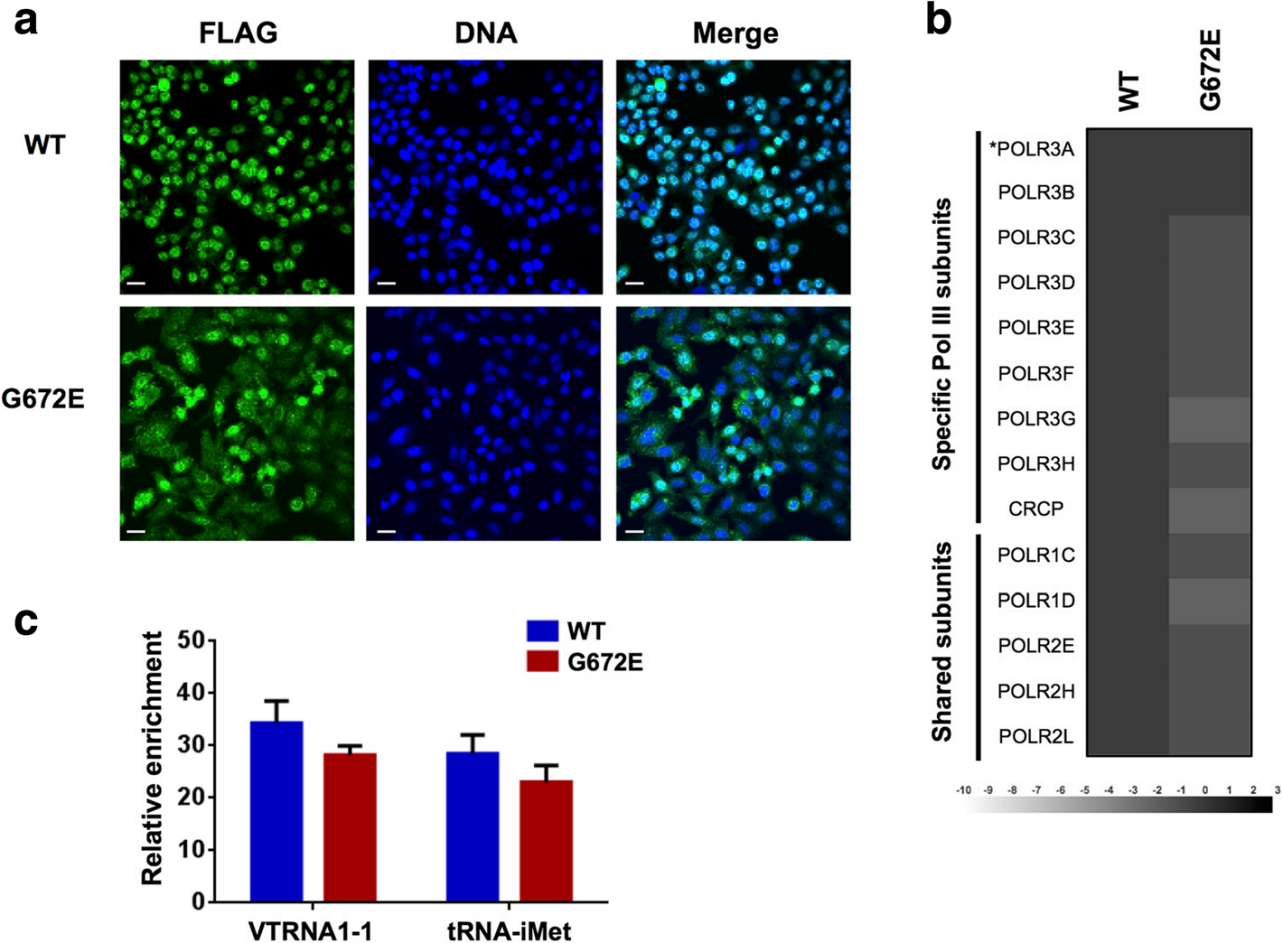
Impact of POLR3A G672E mutation on Pol III function in human cells. **a**) Immonufluorescence experiment showing the predominant nuclear localization of FLAG-tagged variants of POLR3A (WT or G672E). Scale bar = 20 μm. **b**) FLAG-tagged variants of POLR3A (WT or G672E) were expressed at equivalent levels in HeLa cells and purified using anti-FLAG affinity chromatography. The co-purified proteins were identified by LC-MS/MS. The heatmap contains the log_2_-transformed average spectral count ratios of G672E/WT across both replicates. Spectral counts were computed with Mascot. Specific and shared (with Pol I and/or Pol II) subunits are identified on the left. POLR3A (the bait) is identified by an asterisk. **c**) ChIP-qPCR performed against FLAG-tagged variants POLR3A-WT and POLR3A-G672E expressed transiently at equivalent levels in HEK293 cells. The chromatin was quantified by qPCR with primers for two Pol III target gene promoters (VTRNA1-1 and tRNA-iMet). Pol III enrichment at these loci was calculated relative to a locus on chromosome 13 that is not bound by Pol III. Data are represented as mean +/− SEM of biological triplicates

Because of the absence of dysfunction resulting from the c.2015G > A (p.G672E) mutation in mouse, we sought to evaluate its impact on Pol III function in human cells. We stably expressed FLAG-tagged versions of WT and mutant (G672E) POLR3A in HeLa cells. We first examined the impact of the G672E mutation on POLR3A cellular localization by performing anti-FLAG immunofluorescence. As expected, POLR3A-WT showed a predominant nuclear localization. Similarly, the majority of POLR3A-G672E was also in the nucleus, albeit with slightly more of the protein in the cytoplasm compared to WT. This suggests that the mutant Pol III complex is generally correctly assembled and imported into the nucleus (Fig. 5a). To further confirm this, we performed anti-FLAG affinity purification on cell extracts from cell lines expressing FLAG-tagged POLR3A-WT and POLR3A-G672E and analyzed the purified proteins using shotgun proteomics. The mutant POLR3A-G672E subunit was able to pull down all detectable Pol III subunits with levels that did not significantly differ from the WT subunit, indicating that the Pol III complex assembles correctly and thus that the mutation does not globally impair Pol III complex assembly (Fig. 5b, Additional file 1: Table S1). Finally, we performed chromatin immunoprecipitation followed by quantitative PCR (ChIP-qPCR) to evaluate Pol III occupancy at two target loci after transient transfection of POLR3A-WT or POLR3A-G672E in HEK293 cells. This showed a mild reduction in Pol III occupancy for POLR3A-G672E compared to POLR3A-WT, but the difference was not statistically significant (Fig. 5c). These results suggest that the impact of the *POLR3A* c.2015G > A (p.G672E) mutation on Pol III function is also mild in human cultured cells.

## Discussion

In this study, we describe the first transgenic mice with bi-allelic mutations in *Polr3a*, encoding the largest subunit of Pol III. We report that these mice do not display gross hypomyelination or cerebellar atrophy. In fact, our data shows apparently normal myelin staining and myelin protein levels in KI/KI and KI/KO mice at 90 days of age, thereby excluding the presence of hypomyelination in these mice. Furthermore, we did not find evidence of demyelination, gross cerebellar atrophy or Purkinje cell loss in the brains of mice at one year of age. This is consistent with the absence of statistically significant motor dysfunction in one-year-old *Polr3a* KI/KI and KI/KO mice. Altogether, our results are in stark contrast with the observations in the majority of human patients affected with POLR3-HLD described to date, who manifest diffuse hypomyelination and cerebellar atrophy on MRI, childhood-onset ataxia often leading to loss of gait and speech, and death in adolescence or early adulthood [5].

This first report of a missense mutation in subunits of Pol III in a vertebrate model organism thus demonstrates that bi-allelic mutations in *Polr3a* do not necessarily lead to leukodystrophy and/or cerebellar dysfunction in mice, and that Pol III vulnerability to mutations may vary between species. Indeed, a previous report showed that a splice site substitution in zebrafish *polr3b,* leading to an in-frame deletion of 41 amino acids, resulted in impaired intestinal and exocrine pancreas development in the larvae, with no CNS or myelination defects reported [38]. Importantly, instead of the expected 50% decrease in POLR3A protein level in KI/KO mice, we observed normal levels of the full-length protein. This may be due to a compensation mechanism that allows overcoming the loss of one allele by maintaining normal levels of full-length protein, but further experiments are warranted to establish whether this is true. Of note, in one deceased POLR3-HLD patient carrying a heterozygous nonsense mutation in *POLR3A*, there were only 26.8% and 6.8% decreases in POLR3A protein levels in the white matter and the cortex, respectively, compared to a healthy control [1]. This is in agreement with our observation that a heterozygous premature stop codon in *POLR3A* does not necessarily lead to a 50% loss of full-length protein. Furthermore, this may account for the fact that the KI/KO mice are not more severely affected than KI/KI mice, but does not explain the lack of myelin-related phenotype in G672E mutant mice. At the molecular level, we show that the levels of five Pol III transcripts are largely unaffected in the cerebrum and liver of *Polr3a* KI/KI and KI/KO mice, although there was a trend towards a small decrease in precursor tRNA-Ile levels in one-year-old KI/KO mice. While this is consistent with the absence of a clinical or histological phenotype, it surprisingly implies that certain Pol III mutants can function well enough to maintain overall normal levels of Pol III transcripts and general homeostasis in mice. Of note, we cannot exclude effects on the expression of other Pol III targets, especially since Pol III-transcribed genes vary in their promoter structure and associated transcription machinery [39]. Recently, the Pol III transcriptome was investigated in the blood of patients with a homozygous *POLR3A* splice site mutation. This mutation produces an aberrant *POLR3A* mRNA, which is reduced in abundance by 37% relative to the wild-type mRNA [21]. In addition to the technical limitations of assessing tRNA levels in a heterogeneous cell population such as blood, the results suggest that there is only a modest defect in Pol III function in these patients, with 7/46 tRNA isoacceptors showing statistically significant changes. Furthermore, the study reports an increase in the levels of 5S rRNA, RMRP and RPPH1 in patients [21], which is difficult to reconcile with a reduced level of functional POLR3A protein. Investigation of Pol III transcript levels in skin fibroblasts of a classical POLR3-HLD case did not uncover differences in 7SL RNA levels between the patient and a control [14], but this could be due to the fact that fibroblasts, just as blood, are not affected in the disease. Levels of Pol III transcripts were not analyzed in the brain of two deceased POLR3-HLD patients [5, 33].

To better understand the phenotypic discrepancy between human POLR3-HLD cases and our mutant mice, we analyzed the impact of the POLR3A G672E mutation on Pol III function in human cells, as was previously done for leukodystrophy-causing *POLR1C* mutations [3]. Our results suggest that the effect of the POLR3A G672E mutation is milder than the aforementioned POLR1C mutations. In fact, we show that the Pol III complex containing the FLAG-tagged POLR3A-G672E is properly assembled and has a predominant nuclear localization. This is in contrast to the severe complex assembly defect and cytoplasmic localization of both reported POLR1C mutants [3]. Although we observed a mild reduction in Pol III occupancy on chromatin for POLR3A-G672E, this was much more pronounced for the POLR1C mutants. Some of the difference may be explained by the different techniques used (ChIP-qPCR vs. ChIP-Seq, transient vs. stable transfections) [3]. Thus, we cannot exclude that a more substantial defect in Pol III occupancy could be uncovered for POLR3A-G672E using genome-wide techniques. Furthermore, it is possible that the G672E mutation impairs downstream processes such as transcriptional elongation or termination, but this could not be evaluated in the transfected cells since they still express the endogenous POLR3A. The mild impact of the G672E mutation in human cells perhaps explains why this mutation is viable in the homozygous state in humans, and appears to be in agreement with the milder phenotype observed in a subset of patients with this mutation [1].

The human and mouse POLR3A proteins share 97.99% sequence identity and the region surrounding the G672E mutated site (20 amino acids on each side) is perfectly conserved (multiple protein alignment by Clustal Omega [40]). The lack of a strong phenotype in *Polr3a* KI/KI and KI/KO mice could potentially be explained by the much higher proportion of white matter in the human brain (more than 50%) compared to other species (around 10% in mouse) [41–43]. This might make the human brain more vulnerable than the mouse brain to mutations in genes important for myelination. Furthermore, oligodendrogliogenesis is thought to occur at a different pace in humans and mice, perhaps leading to differences in susceptibility to myelin abnormalities [44]. In fact, the disruption of genes causing leukodystrophies in humans does not always produce the same phenotype in mouse. Mouse models have been published for a number of leukodystrophies, but in several of them, the CNS myelin defects are milder than in humans or even completely absent. For example, null mice for *Cx47* or homozygous *Cx47* ^*M282T/M282T*^ mice show only mild myelin deficits, contrary to human patients with mutations in its human ortholog, *GJC2*, which causes Pelizaeus-Merzbacher-like disease [43, 45]. Inactivation of *Abcd1* in mice, associated with human adrenoleukodystrophy (ALD), leads to a late-onset neurological phenotype that resembles adrenomyeloneuropathy, with abnormal myelin and axonal loss in the spinal cord and the sciatic nerve, but these mice do not display the cerebral demyelination characteristic of cerebral ALD [46].

Another possible explanation for the absence of a phenotype in mice is the existence of primate-specific Pol III transcripts. The best example is BC200 RNA, a brain-specific Pol III transcript that is thought to regulate subcellular translation in dendrites [47]. BC200 is only present in primates. Although there is a functional analog (Bc1) in mouse, Bc1 and BC200 have different evolutionary origins [48]. While we did not observe differences in Bc1 levels in KI/KI and KI/KO mice, we cannot exclude the possibility that BC200 may be sensitive to Pol III mutations and have a unique function in the human CNS that is not recapitulated by Bc1 in the mouse. In this scenario, the brain-specific expression of BC200 could explain the mainly CNS manifestations of POLR3-HLD. In recent years, several novel Pol III transcriptional units have been discovered in the human genome. Some of these transcripts are specifically expressed in neuronal cell lines where they have been found to regulate alternative splicing, proliferation, differentiation or cell cycle progression [49–54]. Although functional homologs have been identified in the mouse genome for some of these transcripts, they are not orthologous to their human counterparts. Deregulation of such transcripts could be responsible for the phenotype in humans, but their absence or different evolutionary origin in rodents would not lead to the same manifestations in mice.

The phenotypic spectrum of POLR3-HLD in humans is wide, regarding severity, age of onset and nature of symptoms [5]. Among individuals homozygous for the c.2015G > A (p.G672E) mutation, the disease severity is highly variable, even within families, with 2/5 cases still ambulatory in early adulthood (unpublished data). One possibility is that *Polr3a* KI/KI and KI/KO mice, which carry the same c.2015G > A (p.G672E) mutation, more closely resemble these milder cases. On the other hand, these patients still displayed hypomyelination on MRI [1], while our histology data shows normal myelination in our mice. A recent study described patients with mutations in *POLR3A* or *POLR3B* without hypomyelination, suggesting that this feature is not obligate for POLR3-related disorders [8]. However, cerebellar atrophy and corresponding clinical symptoms were evident in these individuals [8], contrary to the observations in *Polr3a* KI/KI and KI/KO mice. Nonetheless, it is important to note that our experiments were aimed at detecting major differences in the transgenic mice compared to WT mice. The LFB and Nissl stains and immunoblots we performed do not exclude the possibility of altered myelin ultrastructure or physiological dysfunction of Purkinje cell neurons [55, 56]. It remains possible that KI/KI and KI/KO mice will develop later-onset phenotypic abnormalities due to mild pathological mechanisms, but uncovering these events is beyond the scope of the present study, which focused on establishing whether KI/KI and KI/KO mice represent a good model for childhood-onset POLR3-HLD. Nevertheless, the important phenotypic heterogeneity observed in POLR3-HLD suggests the existence of additional genetic or epigenetic factors that can modify the presentation of the disease. The presence of certain genetic variants may be necessary in order to develop a severe form of the disease. If this is the case, introducing the *Polr3a* KI mutation in different mouse backgrounds could lead to a neurological phenotype. Mouse genetic backgrounds often influence the severity of a gene KO or KI [57, 58], and comparison of the same mutation in different strains could allow identification of genetic modifiers [59]. Environmental stressors could also accentuate the disease presentation. This is the case in Vanishing White Matter (VWM) disease, which is caused by mutations in genes encoding the eukaryotic translation initiation factor 2B [60]. An increasing amount of evidence suggests that the expression of different pools of tRNAs is important for protein homeostasis under normal and stress conditions [61–63]. One can imagine that POLR3-HLD cases would be more susceptible to certain stressors during CNS development because of Pol III dysfunction, and those would vary among individuals. Since laboratory mice are housed in a relatively stress-free and sterile environment, exposure to environmental stressors during early development might produce a more severe phenotype.

To our knowledge, even the most mildly affected POLR3-HLD patients manifest some aspects of the disease. Thus, the lack of a phenotype in our mutant mice may not solely be explained by the effects of genetic modifiers or environmental stressors. Perhaps a combination of these or other factors discussed herein could account for the normal CNS development of mice carrying the *Polr3a* c.2015G > A (p.G672E) mutation. This mutation was chosen for this mouse model based on its frequency in the French Canadian patient population and the viability of homozygous carriers [1, 5]. In light of our results, and considering the genetic and phenotypic heterogeneity in POLR3-HLD, it is possible that other *POLR3A*, *POLR3B* or *POLR1C* mutations may produce a more severe phenotype in mice. Thus, the choice of future mutations for insertion into mice should also consider the location of the mutation within important structural elements, such as the bridge helix or the trigger loop, and mutations known to have an impact on Pol III function [22]. Previous studies have found that non-lethal point mutations in conserved regions of yeast Pol III subunits, including two POLR3-HLD-causing mutations, impair Pol III transcription [23, 64–66]. Similar studies on a range of mutations in yeast could aid in selecting mutations to introduce in mice [23]. Alternatively, expression of different mutated forms of Pol III subunits in HeLa cells, as performed with G672E, could also be a helpful tool to choose appropriate mutations. For instance, mutations that cause the accumulation of POLR3A in the cytoplasm may result in a more severe phenotype in mice. However, there is a need for caution since most POLR3-HLD mutations have not been reported in the homozygous state and might be lethal.

Hypomyelination in humans may be due to minor differences in Pol III activity that do not produce the same effect in mice because of a higher tolerance to mutations affecting myelin formation. On the other hand, more severe mutations, such as the ones that are only observed as compound heterozygotes in humans or that cause accumulation of the mutated subunit in the cytoplasm, may be too severe as homozygous alleles and involve other organs or cause developmental failure or embryonic lethality. Nonetheless, both POLR1C mutations that were found to impair complex assembly and nuclear import were present in the homozygous state in patients [3], suggesting that these two features are compatible.

In conclusion, this study illustrates the challenges of developing mouse models for HLD. However, the phenotypic and genetic heterogeneity characteristic of POLR3-HLD patients raises the possibility that introducing other mutations in genes encoding Pol III subunits could lead to a more severe early onset phenotype.

## Methods

### Animals

All experiments were carried out according to good practice of handling laboratory animals consistent with the Canadian Council on Animal Care and approved by the University Animal Care Committee. *Polr3a* ^*KI/KI*^ mice were generated by Ozgene (Bentley, Australia) on a C57BL/6 J genetic background using a conditional mutagenesis strategy modeled after the FLEX switch (see Additional file 1) [67]. To generate whole-body *Polr3a* ^KI/+^ mice, *Polr3a* ^FL/+^ mice were crossed with transgenic mice expressing *CMV-Cre* (Jackson Laboratory #006054). *Polr3a* ^KI/+^ mice were bred together to obtain homozygous *Polr3a* ^KI/KI^ mice (KI/KI). Full-body *Polr3a* ^+/−^ mice were obtained from the Riken Bioresource Center (#RBRC03817, strain B6D2F1-Polr3a < Gt (LVtrap1)LG04Osb>), where they were produced by gene trap (Additional file 1: Figure S2). To produce *Polr3a* ^KI/-^ mice (KI/KO), we bred KI/KI mice with *Polr3a* ^+/−^ mice. The resulting KI/KO mice were bred with KI/KI mice to generate litters with the predicted 50% KI/KI and 50% KI/KO mice.

### Behavioral tests

Mice were tested for general locomotion, balance, coordination and strength. We generated a cohort of 15 female mice per group (WT, KI/KI, KI/KO), all born within seven days of one another, and submitted them to the following behavioral tests over one year. Phenotyping tests were performed at 40, 90 and 270 days of age. The balance beam was also repeated at 365 days of age, while the gait analysis was done at 270 and 365 days old. The balance beam, rotarod and inverted grid tests were performed as previously reported [68, 69]. For the open field test, locomotor activity was assessed over 90 min in a bank of 8 Versamax Animal Activity Monitor chambers (Accuscan Model RS2USB v4.00, Columbus, OH). Each chamber consisted of a clear acrylic open-field (40 cm L × 40 cm W × 30 cm H), divided into two equal sized chambers (20 cm L × 20 cm W × 30 cm H) by an acrylic partition and was covered by an acrylic lid with air holes. Activity was detected via a grid of infrared photo sensors spaced 2.5 cm apart and 6 cm above the floor along the perimeter of the box. All activity chambers were connected to a Versamax data analyzer (Accuscan Model VMX 1.4B, Columbus, OH), which then transmitted data to an HP Compaq Pentium 4 computer for further analysis. Locomotor activity and its distribution within the two chambers were quantified using the Versamax Software System (Version 4.00, Accuscan, Columbus, OH). The following measures were recorded for each interval of 10 min: total distance covered, number of movement bouts, time moving, stereotypy bouts and stereotypy time. For gait analysis, which was performed at 270 and 365 days old only, we used footprint patterns or walking tracks to analyze different parameters [70]. The fore and hind paws of the mice were stained with red and blue washable color paint, respectively and the mice were trained to walk on a paper-covered narrow runway (85 cm long, 6 cm wide with clear Plexiglas walls) until they reached a dark box at the end of the runway. If the animal stopped in the middle of the track, the test was repeated. The first and last 10 cm of the footprint were excluded. For analyses, at least four steps from each side per print were measured. Stride length, front and hind limb width and inter limb coordination were measured bilaterally. Upon completion of the last tests, 1-year-old mice were sacrificed and tissues were harvested for histology or for RNA extraction (see below).

### Histology

For tissue preparation, mice were anesthetized with mouse anesthetic cocktail (ketamine (100 mg/ml), xylazine (20 mg/ml) and acepromazine (10 mg/ml)), perfused transcardially with 0.9% NaCl followed by 4% paraformaldehyde. Brains were dissected and post-fixed for 24 h at 4 °C in the same fixative. For Luxol Fast Blue (LFB), tissue processing, embedding, sectioning and staining were performed at the Goodman Cancer Research Centre Histology Facility (McGill University, Montreal, Canada). Briefly, tissues were embedded in paraffin and sectioned on a microtome. Sections of 15 μm were stained with LFB according to standard procedures. Nissl stains and Purkinje cell counts were performed as previously reported [71].

### Western Blots

Cerebellar or cerebral hemispheres were harvested, snap-frozen in liquid nitrogen and homogenized with a Teflon putter in extraction buffer [10 mM Tris–HCl, pH 7.5, 150 mM NaCl, 1 mM 6 EDTA, 1% Triton X-100 and protease inhibitors (Roche)] followed by centrifugation at 12,000xg for 30 min and collection of the supernatant. Protein quantification was determined using DC Protein assay (Bio-Rad). Protein samples were separated onto a 4-12% NuPAGE Bis Tris gel (ThermoFisher) and transferred onto a nitrocellulose membrane (Bio-Rad). For assessment of myelin protein levels, immunoblots were probed with anti-MBP (Aves Labs Inc. #MBP), anti-PLP (Abcam #ab28486), anti-CNPase (Millipore, #MAB326R), anti-MAG (gift of Dr. David Colman, McGill University) and anti-tubulin (Sigma-Aldrich #T5168) primary antibodies. For measurement of POLR3A protein levels, immunoblots were probed with anti-POLR3A (Abcam #ab96328) and anti-actin (Abcam #ab3280).

### RNA extraction and Northern Blots

Cerebral hemispheres and livers were harvested and snap-frozen in liquid nitrogen. Of note, one-year-old mice were fed *ad libitum* for their entire life, while 90-days-old mice used for Northern Blots were fasted for 16 h and refed for 5 h prior to sacrifice and tissue collection in order to stimulate Pol III transcription. Tissues were homogenized in Qiazol lysis reagent (Qiagen). Total RNA was extracted with the miRNeasy kit (Qiagen) and treated with DNAse I (Qiagen) according to the manufacturer’s instructions. RNA quality was assessed on an Agilent 2100 Bioanalyzer and RNA Integrity Numbers (RIN) were routinely above 9. For Northern Blots, RNA samples (7.5ug or 10 ug) were separated by denaturing polyacrylamide gel electrophoresis and transferred to Nytran Plus membranes (GE Healthcare). The resulting blots were sequentially hybridized with [^32^P]-end labelled probes detecting precursor tRNA^Ile(TAT)^, mature tRNA^Leu(AAG)^ and mature tRNA^Glu(CTC)^ at 42 °C. Bc1 RNA levels were subsequently detected with a probe mapping to the 5′ portion of the RNA, as previously described[72]. For n-Tr20, blots were sequentially hybridized with a probe targeting the 3′ trailer sequence of precursor n-Tr20 and a probe targeting both precursor and mature n-Tr20 [37]. All Pol III transcript levels were quantified and normalized to U3 snRNA levels.

### Immunofluorescence, affinity purification and mass spectrometry

HeLa cell lines stably expressing the FLAG-tagged POLR3A subunit (WT or G672E-mutated) were generated by transfection with Lipofectamine according to the manufacturer’s instructions (ThermoFisher). Immunofluorescence was performed using an anti-FLAG antibody, as previously described [3]. For affinity purification, cytoplasm and nuclei were prepared as reported before [73]. Briefly, cells were lysed by mechanical homogenization in lysis buffer [10 mM Tris–HCl (pH 8), 0.34 M sucrose, 3 mM CaCl2, 2 mM MgOAc, 0.1 mM EDTA, 1 mM DTT, 0.5% Nonidet P-40 and protease inhibitors]. Whole cell extracts were centrifuged at 3,500 × g for 15 min and the supernatant, which represents the cytoplasmic fraction, was saved. The pellet containing the nuclei was resuspended, lysed by mechanical homogenization in lysis buffer [20 mM HEPES (pH 7.9), 1.5 mM MgCl2, 150 mM KOAc, 3 mM EDTA, 10% glycerol, 1 mM DTT, 0.1% Nonidet P-40 and protease inhibitors] and, centrifuged at 15,000 × g for 30 min. The supernatant, which corresponds to the nucleoplasmic fraction, was saved. Cytoplasm and nuclei were mixed; fractions were centrifuged at 124,000 × g and dialyzed overnight in dialysis buffer [10 mM Hepes (pH 7.9), 0.1 mM EDTA (pH 8), 0.1 mM DTT, 0.1 M KOAc and 10% glycerol]. The following day, the fractions were clarified by centrifugation at 20,000 × g for 30 min, and the supernatants containing the solubilized proteins were collected. Anti-FLAG affinity purification and mass spectrometry were performed as previously reported [3]. Data analysis is described in Additional file 1: Supplementary Methods.

### ChIP-qPCR

For ChIP-qPCR, FLAG-tagged POLR3A variants (WT or G672E) were transiently transfected in HEK293 cells for 24 h with Lipofectamine. Transfections were performed in triplicate. Cells were crosslinked with 1% formaldehyde directly in the cell medium for 5 min followed by quenching for 5 min in 125 mM glycine. ChIP was performed as reported previously [3]. For qPCR, 10 ng of chromatin were used to amplify two Pol III target genes (*VTRNA1-1* and *tRNA-iMet*) and a control locus on chromosome 13 that is not bound by Pol III. The following primers were used: *VTRNA1-1*: 5′-GGC TGG CTT TAG CTC AGC G-3′ and 5′- TCT CGA ACA ACC CAG ACA GGT-3′, *tRNA-iMet*: 5′-AGA GTG GCG CAG CGG AA-3′ and 5′- TAG CAG AGG ATG GTT TCG ATC C-3′, unbound locus: 5′-GGC ACT GTC TTG TCA CTG CAC ATT-3′ and 5′- TGG AAA CAG CCA TTG AGA ACA CC-3′.

### Statistical analyses

Data are presented as mean +/− SEM. Preliminary analysis revealed significant differences in weight among the genotypes, particularly at the later ages (see Additional file 1: Figure S3). As weight can significantly impact motor performance, behavioral measures at each age were examined by one-way analysis of covariance (ANCOVA) with weight as the covariate. Behavioral measures are reported as ANCOVA (weight) adjusted least squares means +/− SEMs in Fig. 1. Unadjusted means +/− SEMs are shown in Figure S4 (see Additional file 1). Purkinje cell counts and RNA quantifications were compared using one-way ANOVA. ANOVAs were performed with GraphPad Prism and ANCOVAs with JMP (Version 13). In all cases, the threshold for statistical significance was set at *p-*value < 0.05.

## Additional file

Additional file 1:
**Figures S1.** to **S6**, **Table S1.** and Supplementary Methods (PDF 7816 kb)

## Abbreviations

cDNA: Complementary DNA
CNS: Central nervous system
HLD: Hypomyelinating leukodystrophy
KI: Knock-in
KO: Knock-out
LFB: Luxol Fast Blue
MRI: Magnetic resonance imaging
MS: Mass spectrometry
ChIP: Chromatin immunoprecipitation
ncRNA: Non-coding RNA
Pol III: RNA Polymerase III
rRNA: Ribosomal RNA
SEM: Standard error of the mean
tRNA: transfer RNA
WT: Wild-type
