# Supplementary material for: Absence of neurological abnormalities in mice homozygous for the Polr3a G672E hypomyelinating leukodystrophy mutation
Source: Mol Brain. 2017 Apr 13;10:13. doi: 10.1186/s13041-017-0294-y (PMC5391615; doi:10.1186/s13041-017-0294-y)
Supplement: Additional file 1: — Figures S1. to S6, Table S1. and Supplementary Methods (PDF 7816 kb) [file 13041_2017_294_MOESM1_ESM.pdf]

Additional File 1: Supplementary Material

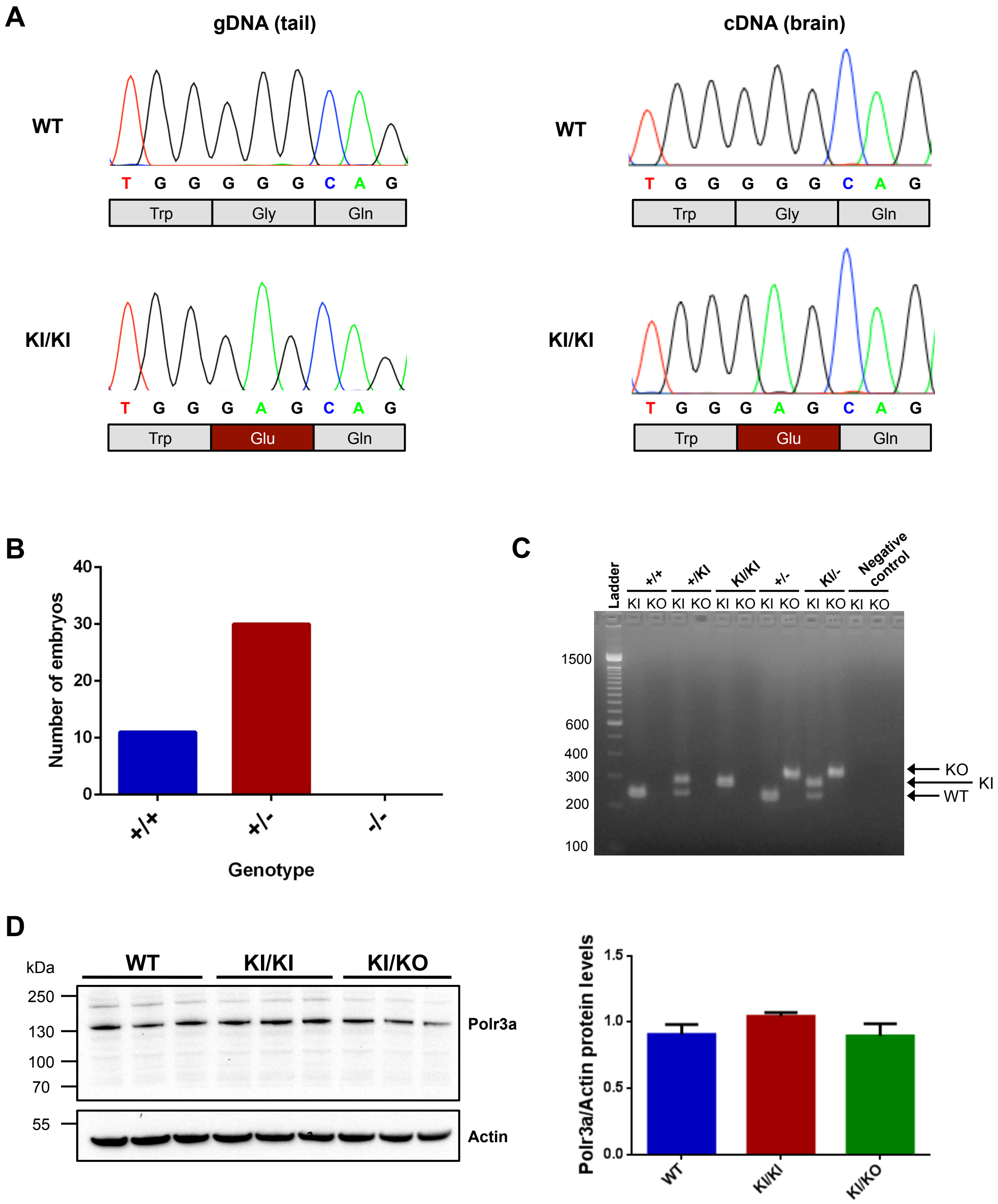

Figure S1. Generation of *Polr3a* transgenic mice. A) Sequence chromatograms of genomic DNA (left) and complementary DNA (right) for a WT mouse and a *Polr3a* KI/KI mouse, showing the presence of the homozygous mutation c.2015G>A (p.G672E) in the KI/KI mice. B) Genotyping of pups from *Polr3a*$^{+/-}$ intercrosses shows that the *Polr3a*$^{-/-}$ genotype is not compatible with embryo development. Embryos were collected at E13.5 from six pregnancies. Genotyping of embryos shows

normal ratio of *Polr3a*$^{+/+}$ and *Polr3a*$^{+/-}$ mice but no *Polr3a*$^{-/-}$ mice could be obtained. C) Agarose gel (3%) showing the PCR products from the amplification of genomic DNA for genotyping of *Polr3a* WT, KI and KO alleles. For each mouse, two PCRs were performed and resolved side by side: the first one detects WT and KI alleles and the second detects the KO allele. gDNA: genomic DNA; cDNA: complementary DNA. D) Immunoblot of POLR3A protein levels using protein extracts from the cerebrum of 1-year-old WT, KI/KI and KI/KO mice (n=3 per group). Actin was used as a loading control. Quantification of POLR3A protein levels normalized to Actin levels is shown on the right. Data are represented as mean +/- SEM.

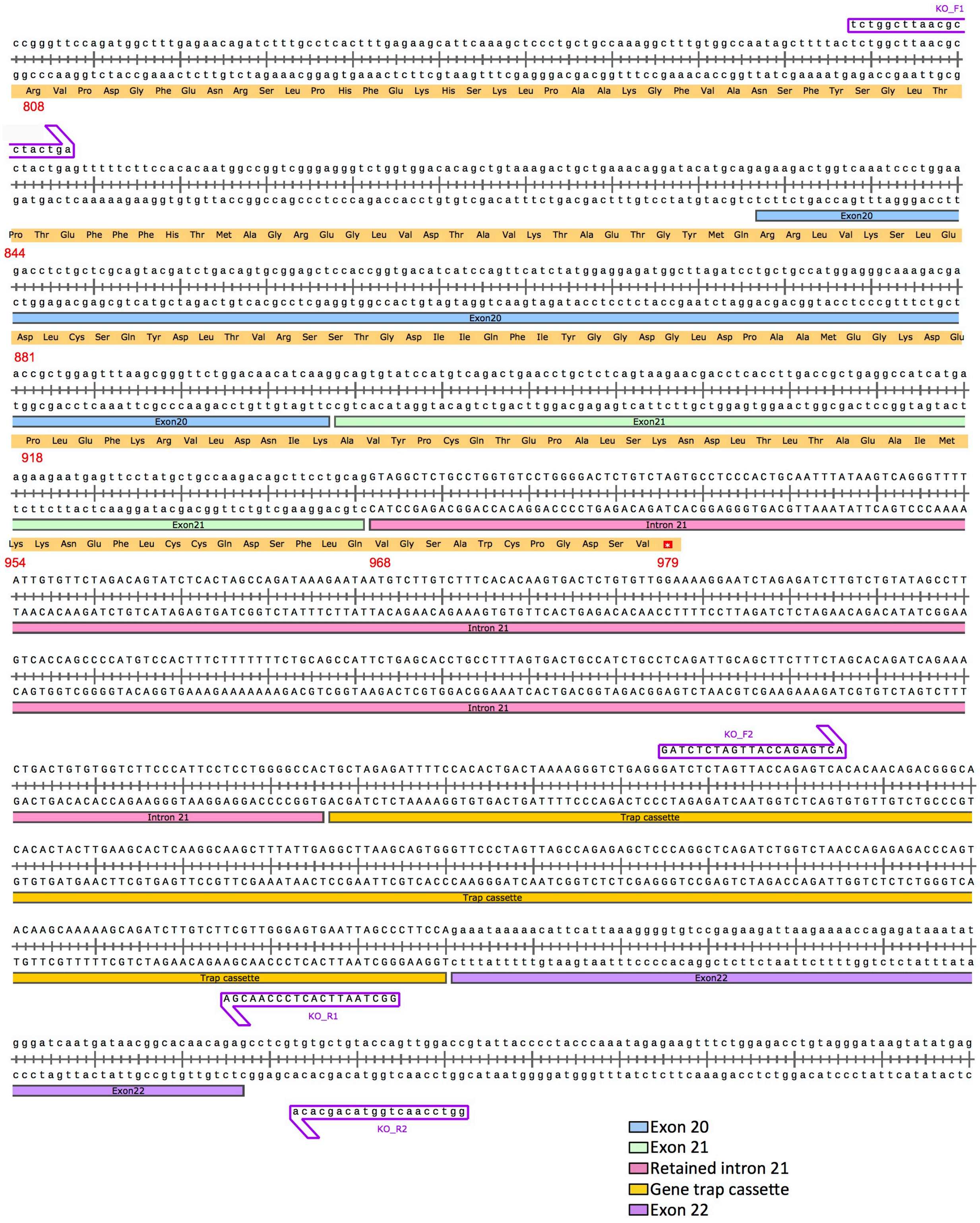

Figure S2. Representation of the *Polr3a* KO allele at the mRNA level. Screenshot of the SnapGene sequence file corresponding to exons 20 to 22 of the *Polr3a* KO mRNA, showing the retention of a portion of intron 21, leading to a frameshift and premature stop codon (red asterix). Open reading frame is shown below the nucleotide sequence. Amino acid numbers are indicated in red. Purple arrows show the primers used for PCR amplification and sequencing.

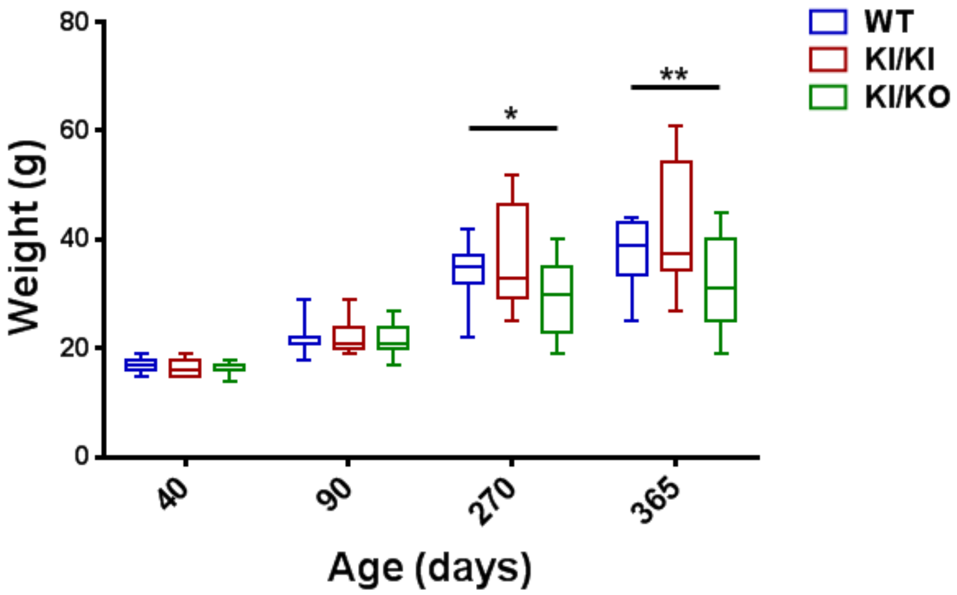

Figure S3. Weights of mice undergoing phenotypic tests at each time point tested. Boxplots show the variability in weight, especially in the mutant groups.

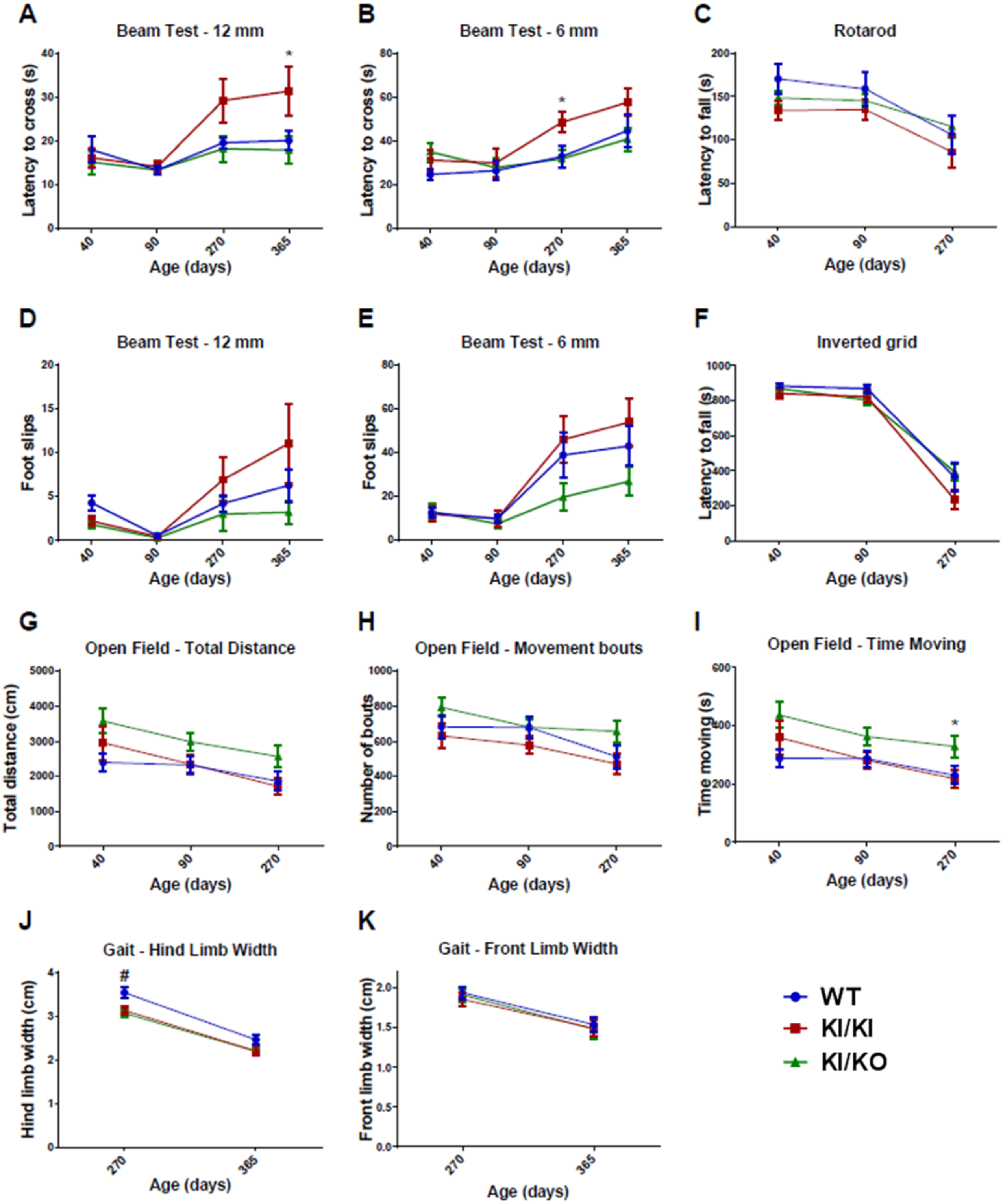

Figure S4. Behavioral data prior to adjustment with weight as covariate (see Figure 1). Results from the 12mm (A, D) and 6 mm (B, E) beam test at four time points consisting of three trials per mouse. Latencies to cross (A, B) and number of foot slips (D, E) were recorded for both beam sizes. C, F) Results from the rotarod (C) and inverted grid (F) tests performed at three time points. The rotarod and inverted grid consisted of three trials per mouse. G-I) Results from the open field test performed at

three time points. The open field test was run for 90 minutes per mouse during which total distance traveled (G), number of movements bouts (H) and total time spent moving (I) were recorded for each 10 minute interval. The results represent the sum of all 10 minutes intervals. J-K) Results from gait analysis performed at the two latest time points. Paws were covered in color paint and mice were allowed to walk on a white paper-covered narrow runway. Distance between fore limbs and hind limbs was measured. All tests were performed on ≥14 female mice per group. For the beam test, rotarod and inverted grid, data are represented as adjusted least squares means +/- SEM of the sum of the three trials for each group. Groups were compared with one-way ANOVA for each time point. *: $p < 0.05$, #: $p < 0.01$.

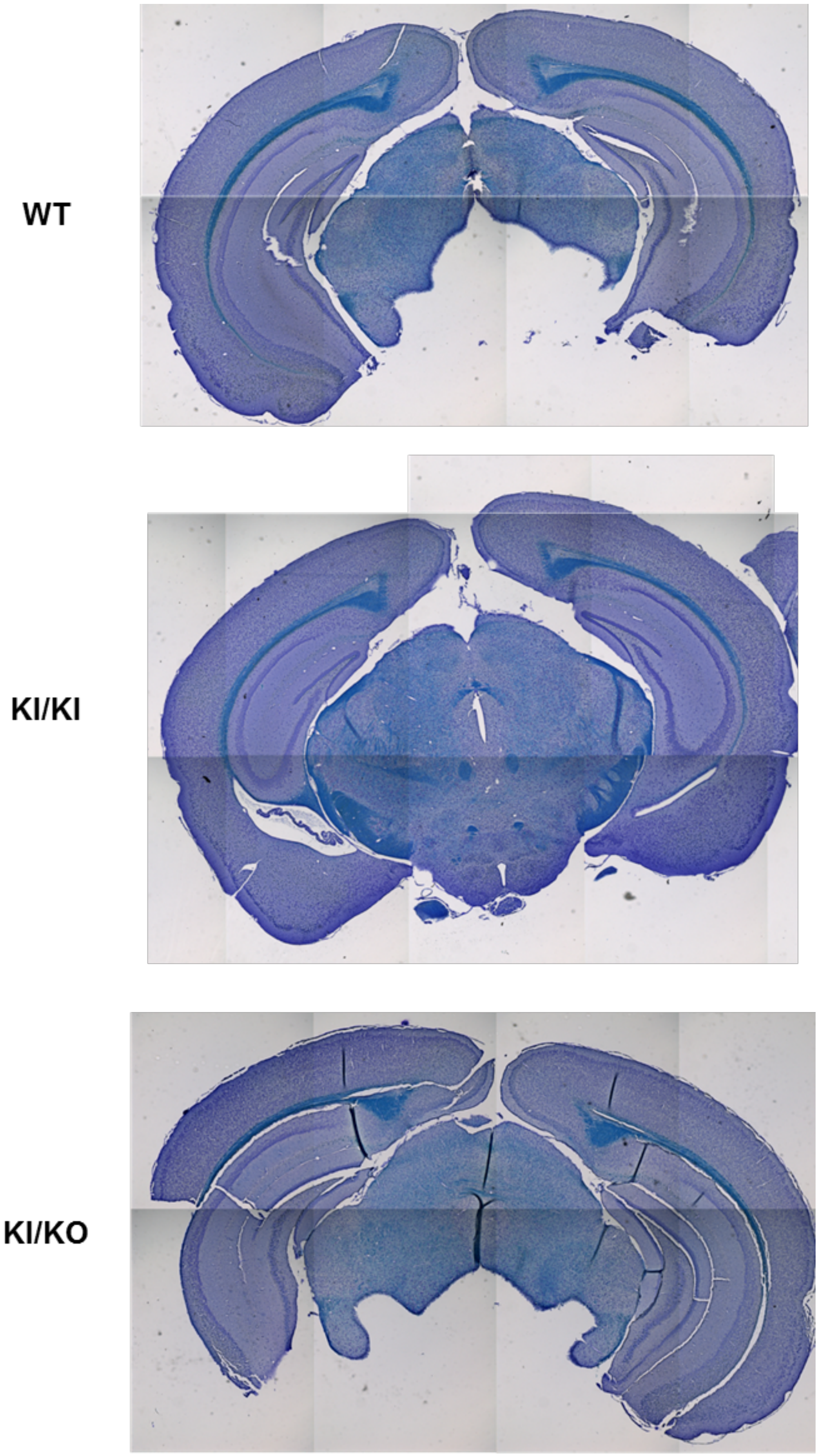

Figure S5. Luxol Fast Blue staining of coronal sections of the cerebrum of 365 days old mice. Staining was performed on four mice per group and representative images are shown for each group. Pictures were taken with the 2.5X objective.

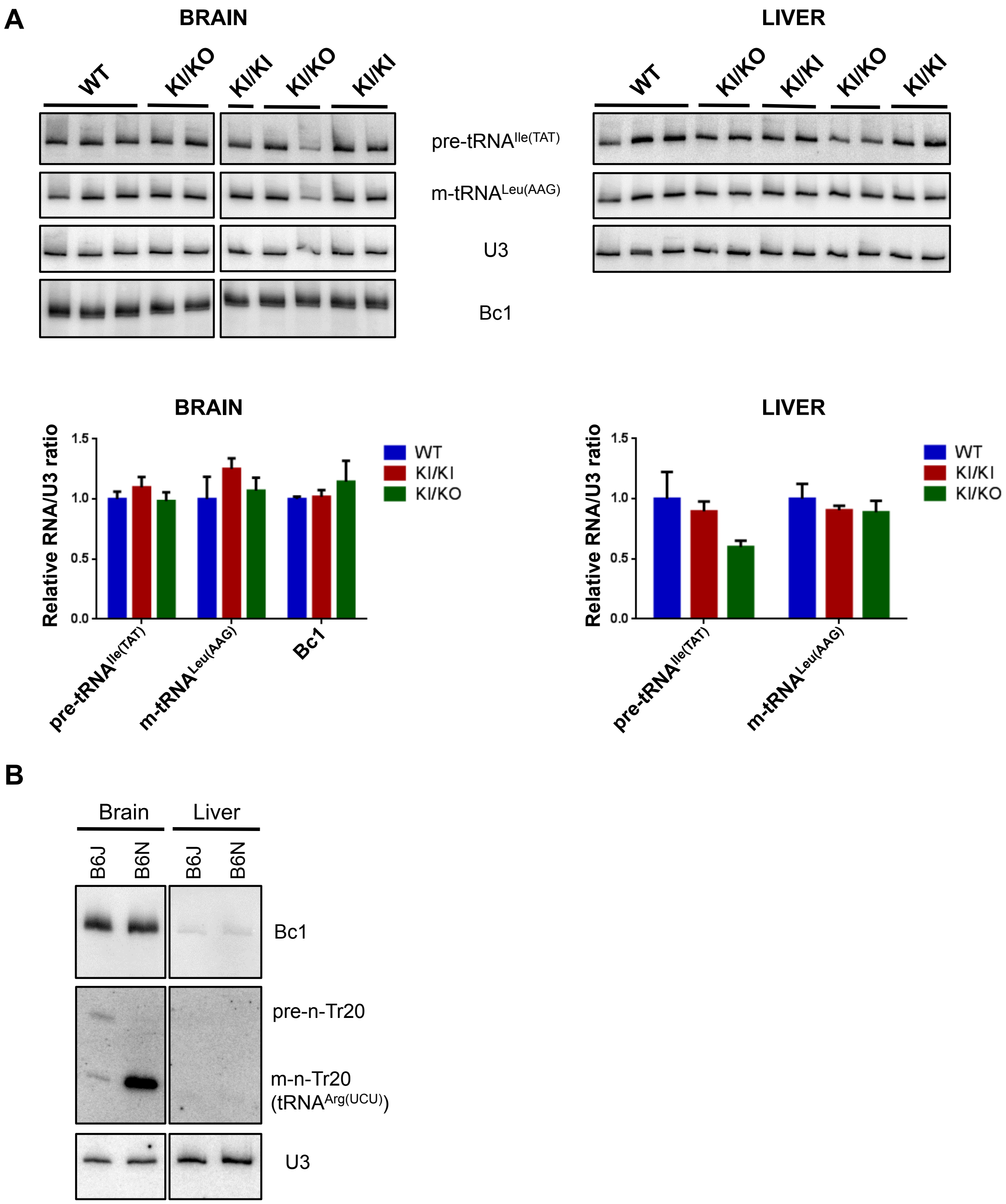

Figure S6. Expression levels of Pol III transcripts in the brain and liver of *Polr3a* KI/KI and KI/KO mice. A) Top: Northern blots of one precursor (pre) tRNA, one mature (m) tRNA and Bc1 RNA from the cerebrum and liver of 90 days old mice. U3 snRNA was used as a loading control. Bottom: Quantification of transcripts surveyed by Northern Blot. Pol III transcript levels were normalized to U3 snRNA levels. No statistically significant differences were uncovered. Data are represented as mean +/- SEM. B) Northern blot of Bc1 and n-Tr20 in the brain and liver of C57BL/6J (B6J) and C57BL/6N (B6N) mice, showing the brain-specific expression of these transcripts, as well as the much higher expression of n-Tr20 in B6N mice. U3 snRNA was used as a loading control.

Table S1. Quantification of the high-confidence interactors of the G672E mutant POLR3A against WT POLR3A. The ratio represents the average of the two spectral count ratios from the duplicate pairs of POLR3A G672E and POLR3A WT. The adjusted p-values were obtained by performing a two-tailed one-sample t-test followed by multiple testing correction using the Benjamini-Hochberg procedure. Specific Pol III subunits are show in blue and shared subunits with Pol I and/or Pol II are indicated in green.

Protein Symbol	Ratio (G672E/WT)	Adjusted p-value
C19orf60	0.500	0.80
CRCP	0.227	0.73
FGD2	0.619	0.81
MAF1	0.378	0.73
PDRG1	1.299	0.97
PFDN2	0.577	0.81
PFDN6	0.224	0.73
PIH1D1	0.993	0.97
POLR1C	0.677	0.81
POLR1D	0.300	0.73
POLR2E	0.559	0.81
POLR2H	0.389	0.73
POLR2L	0.410	0.73
POLR3A	1.000	0.97
POLR3B	0.787	0.89
POLR3C	0.479	0.80
POLR3D	0.622	0.81
POLR3E	0.660	0.81
POLR3F	0.419	0.73
POLR3G	0.333	0.73
POLR3H	0.522	0.80
RFC3	0.250	0.73
RPAP3	1.335	0.97
RUVBL1	1.314	0.97
RUVBL2	1.158	0.97
SPTAN1	0.103	0.73
SSB	0.283	0.73
TRMT1L	0.692	0.81
URI1	1.200	0.97
UXT	0.958	0.97
WDR92	0.792	0.89

Supplementary Methods

Generation of Polr3a *KI mice*

The targeting vector for conditional mutagenesis was modeled on the FLEX switch[1]. Briefly, a lox2272 site was inserted in forward orientation into intron 14 of *Polr3a*, approximately 100bp upstream of the exon 15 splice acceptor. A neomycin (neo) cassette was inserted into intron 15, approximately 150bp downstream of the splice donor. The neo cassette was flanked with FRT sites for removal by Flpe-mediated recombination. The neo cassette was followed by a loxP site in forward orientation. The mutant exon 15 (c.2015G>A, p.G672E) was inserted in reverse orientation 5' to the loxP site. The inverted exon was followed by a FLEX switch consisting of lox2272 and loxP in reverse orientation.

DNA extraction, genotyping and Sanger sequencing

Genomic DNA was extracted from tail biopsies or whole embryos using the Gentra Puragene Tissue Kit (Qiagen). For genotyping, two PCR reactions were performed for each mouse and resolved on a 3% agarose gel (Fig. S1C). For the WT and KI alleles, the following primers were used: 5'-ATC ATC CGG GTG GAA TGT AA-3' and 5'-TAA GTG TGC TCT CCC ACA CG-3', producing bands of 246bp and 286bp for the wild-type (WT) and KI alleles, respectively. For the KO allele, the following primers were used: 5'-GTC ACT CAA TCC TCT GCC TTT G-3' and 5'-GAT CTC TAG TTA CCA GAG TCA-3'. The latter primer is located in the gene trap cassette, thus producing an amplicon of 320bp only when the KO allele is present (Fig. S1C). For Sanger sequencing of *Polr3a* exon 15, genomic DNA was amplified by PCR using the following primers: 5'- GGA TGT AAA CAT TAT TCT CCA CCA G-3' and 5'- ACT AAG CCT TTC CCT CTG CG-3'. PCR products were sequenced at the McGill University and Genome Quebec Innovation Center, using a 3730XL DNA Analyzer

(Applied Biosystems). Sequence chromatograms were analyzed using SnapGene v2.7.2 (GSL Biotech LLC) and 4Peaks (A. Griekspoor and Tom Groothuis, mekentosj.com). For determination of the lethality of homozygous KO mice, heterozygous *Polr3a*$^{+/-}$ mice were interbred and gestating females were sacrificed at E13.5 to extract embryos for genotyping.

RT-PCR and cDNA sequencing

For RT-PCR, brain RNA was reversed transcribed into complementary DNA (cDNA) using the Superscript III Reverse Transcriptase (ThermoFisher) according to the manufacturer's instructions. For sequencing of the c.2015G>A (p.G672E) mutation, exons 14 to 16 of *Polr3a* were amplified by PCR using the following primers: 5'-TCA CCC TCA AGG ACA CCT TC-3'and 5'-CGG ATC ACA GAC AGC TCC TT-3'. For sequencing of the KO allele, exons 20 to 22 of *Polr3a* were amplified by PCR in KI/KO mice using the following primers: KO_F1: 5'-TCT GGC TTA ACG CCT ACT GA-3', KO_F2: 5'-GGC TAA TTC ACT CCC AAC GA-3' (located in the gene trap cassette), KO_R1: 5'-GAT CTC TAG TTA CCA GAG TCA-3' (located in the gene trap cassette) and KO_R2: 5'-GGT CCA ACT GGT ACA GCA CA-3' (see Figure S2). PCR products were Sanger sequenced as described above.

Analysis of mass spectrometry data

Protein database searching and protein spectral count quantification were performed with Mascot (version 2.3.02)[2]. The NCBI_Human protein sequence database was downloaded on 20 February 2014. Known protein contaminants such as keratins, which are not expressed in HeLa cells, were excluded from the data set. Undistinguishable protein isoforms were considered as a single protein. For

each LC-MS/MS analysis, protein spectral counts were normalized by the spectral count of the FLAG-POLR3A in order to allow the comparison of different purifications. Each replicate LC-MS/MS analysis of the affinity purifications of the FLAG-POLR3A mutant (G672E) was paired with a LC-MS/MS analysis of the WT FLAG-POLR3A that was performed at the same time. The set of high-confidence interactors of POLR3A for a given mutant analysis was identified by comparing the spectral counts of the interactors obtained from the purifications of the paired WT POLR3A and the G672E POLR3A to those of the proteins purified with an empty vector (EV) of the FLAG tag (nonspecific interaction). A protein is labeled as a high-confidence interactor if it was identified and quantified in both replicates of WT POLR3A and G672E POLR3A and that the ratio of the average spectral counts across the two replicates (WT/EV or G672E/EV) was greater than 5. These stringent criteria allow us to eliminate the vast majority of nonspecific interactors of POLR3A for the analysis of the interactions of the mutant. For each high-confidence interacting protein, a two-tailed one-sample t-test was performed on the spectral count ratios (G672E/WT) and the resulting p-values were adjusted for multiple hypothesis testing using the Benjamini–Hochberg procedure. To maximize the specificity of our approach, a protein is deemed to show a level of differential interaction with POLR3A that is statistically significant when its adjusted p-value is <0.05.

References

1. Schnutgen F, Ghyselinck NB: **Adopting the good reFLEXes when generating conditional alterations in the mouse genome.** *Transgenic Res* 2007, **16:**405-413.
2. Perkins DN, Pappin DJ, Creasy DM, Cottrell JS: **Probability-based protein identification by searching sequence databases using mass spectrometry data.** *Electrophoresis* 1999, **20:**3551-3567.
